# A Brazilian pulp and paper mill effluent disrupts energy metabolism in immature rat testis and alters Sertoli cell secretion and mitochondrial activity

**DOI:** 10.1590/1984-3143-AR2019-0116

**Published:** 2020-06-26

**Authors:** Vanessa Staldoni de Oliveira, Allisson Jhonatan Gomes Castro, Juliana Tonietto Domingues, Ariane Zamoner Pacheco de Souza, Débora da Luz Scheffer, Alexandra Latini, Carlos Henrique Lemos Soares, Glen Van Der Kraak, Fátima Regina Mena Barreto Silva

**Affiliations:** 1 Departamento de Bioquímica, Universidade Federal de Santa Catarina, Florianópolis, SC, Brasil; 2 Department of Integrative Biology, University of Guelph, Guelph, Ontario, Canada

**Keywords:** testis, Sertoli, effluent, lactate, secretion

## Abstract

Our objective was to investigate whether the pulp and paper mill industry effluent could affect the testis and Sertoli cells in a fast exposure period. For this, the present study was carried out in immature rats at 10-day-old. Testis treated *in vitro* with 4% effluent for 1 h presented changes in energy metabolism in terms of a decrease in lactate content and glucose uptake. Elevation in GSH content, as an antioxidant defense mechanism, was also detected. Sertoli cells treated with 4% effluent for 1 hour showed alterations in the mitochondrial metabolism that favor the decoupling of oxidative phosphorylation and the generation of oxygen reactive species and also a time and concentration-dependent delay secretion of acidic vesicles. Our results showed that pollutants present in the pulp and paper mill effluents, in a short time of exposure, are capable of inducing alterations in important metabolic functions in the testis and in Sertoli cells that are crucial for the correct progression of spermatogenesis and fertility.

## Introduction

The male immature reproductive phase is characterized by marked morphological changes which underlie a critical change in the development of the testis (Mendive et al., 2006). Concerning to the rats, in neonatal period, from postnatal day 8 to 20, the testis consists basically of spermatogonia, Sertoli cells and immature Leydig cells that are in a phase of wide proliferation (Picut and Parker, 2017). The blood-testis-barrier (BTB), an important physical and functional barrier formed by Sertoli cell junctions, controls the passage of molecules into the germinal epithelium (França et al., 2016) and creates an immune-privileged microenvironment (Mruk and Cheng, 2015). In rats, BTB arises between 15 and 25 day-old (Morales et al., 2007). For this reason the immature reproductive phase, prior to the BTB formation, is also a sensitive exposure window for certain toxicants that may affect intra‐testicular environment and adversely disturbs spermatogenesis (Picut and Parker, 2017; Gonçalves et al., 2018).

There is an interesting relationship between the metabolisms of two testicular cells types: Sertoli cell can metabolize different energetic substrates but preferably use glucose, most of which is converted to pyruvate via glycolysis. Pyruvate, in turn, is mainly converted to lactate by the activity of the lactate dehydrogenase enzyme (LDH), and exported to the germ cells, essential for their development (Rato et al., 2012; Liu et al., 2016). Despite being an energy substrate, glucose is not the main metabolite used for ATP synthesis in Sertoli cells, the main source of energetic substrate for them is through the oxidation of fatty acids, a process dependent on the mitochondrial metabolism (Rato et al., 2012). Thus, a healthy mitochondrion is essential for the correct functioning of the Sertoli cell, since it is a cell with a fairly high energy demand (Riera et al., 2009). Besides lactate, Sertoli cells secrete amino acids, carbohydrates, lipids, vitamins, metallic ions, fluid, regulatory growth factors, hormones, among other substances to germ cells. Disturbances in any of these mechanisms of synthesis or secretion of metabolic products from testis cells may lead to male fertility disorders (Rato et al., 2012; Alves et al., 2013; Liu et al., 2016).

Other closely related factors in the etiology of male fertility disorders are oxidative stress (Sedha et al., 2015; Bisht et al., 2017) and disturbances in calcium homeostasis (Cavalli et al., 2013; Gonçalves et al., 2018) in the testis environment. Clinical studies demonstrated a correlative relationship between infertility and oxidative stress in sperm (Agarwal et al., 2014). In the testis, reactive oxygen species (ROS) and reactive nitrogen species (RNS) are generated mainly in mitochondria (Aitken et al., 2016) and can induce lipid peroxidation and DNA fragmentation, thereby disrupting the ability to support normal spermatogenesis (Aitken and Román, 2008). In addition, exposure to environmental toxicants can induce oxidative stress and disrupts the male reproductive health (Agarwal et al., 2014). Calcium, in turn, participates in essential biochemical processes for male fertility as testosterone synthesis (Costa et al., 2010), sperm activation (Blomberg Jensen, 2014), BTB homeostasis (Lyon et al., 2017) and cell secretion (Zanatta et al., 2013). Recent studies have shown the relationship between xenobiotic exposure and testicular disorders through disruption in calcium homeostasis (Gonçalves et al., 2018; Liu et al., 2018; Ham et al., 2019).

The pulp and paper mill industry is considered a major contributor to water pollution and has significantly expanded its activities over the last 25 years (Dey et al., 2013). The effluent from this industry is a complex mixture that may contain many biologically active molecules, including compounds derived from different stages of pulp and paper production such as phenols, chlorinated compounds, furans, dioxins, dyes, bleaching agents, heavy metals and others. The pulp and paper mill effluent also contains natural wood derived compounds such as lignin, tannins, acid resins, terpenoids and phytosterols (Dey et al., 2013). Recent studies show that exposure to pulp and paper mill effluent can cause deleterious effects on fish reproduction such as increased plasma concentrations of vitellogenin in females and males and the presence of intersex characteristics in males of *Onchorhynchus mykiss* (Chiang et al., 2015) and decreased oviposition in females of *Pimephales promelas* (Waye et al., 2014). A recent study from our research group showed that pulp mill effluent may adversely affect testis function of *Danio rerio* fish (Castro et al., 2018). However, there is a lack of studies reporting the impacts of pulp and paper mill effluent on reproduction in other taxonomic groups, such as mammals.

Our hypothesis is that the pulp and paper mill effluent from an industry located in the state of Santa Catarina, Brazil, in a short time of exposure and at an environmentally relevant concentration (that is the concentration of the effluent in the natural environment where it is discharged) may affect biochemical functions of the testes and Sertoli cells from immature mammals, important for the correct development of an active and complete spermatogenesis. For this various biochemical responses of immature 10-day-old male rat testis and Sertoli cells following *in vitro* exposure to pulp and paper mill effluent were examined. It included assessments of changes in the activity of testicular enzymes, the synthesis of metabolic products, testes calcium ion homeostasis, oxidative stress as well as Sertoli cell mitochondrial oxygen consumption and cellular secretory activity. Our goal was to investigate how an industrial residue abundantly released into the environment can alter biochemical functions in the mammal testis and Sertoli cells.

## Materials and methods

### Chemicals

2′,7′-dichloro-dihydro-fluorescein diacetate (H_2_DCFDA) was obtained from Thermo Fisher Scientific (Waltham, MA, USA). Carbonyl cyanide *p*-trifluoromethoxyphenylhydrazone (FCCP), rotenone, antimycin A, Dulbecco′s modified Eagle′s medium (DMEM), Ham′s F12 medium, quinacrine and dihydrorodamine 123 were purchased from Sigma Chemical Co. (St.Louis, MO, USA). [U-^14^C]-2-Deoxy-D-glucose (^14^C–DG, specific activity 10.6 GBq/mmol) and [^45^Ca]CaCl_2_ (specific activity 321 KBq/mg Ca^2+^) were purchased from PerkinElmer (Waltham, MA, USA). All other chemicals were of analytical grade.

### Pulp and paper mill effluent

Effluent was collected in February 2015 from Klabin pulp and paper mill, producer of pulps unbleached, located in Correia Pinto, Santa Catarina, Brazil. Effluent was collected after the last step of treatment and immediately before being released to the Canoas River. Testing of the effluent at a 4% dilution was based on previous studies of the toxicity of effluent from this mill on *D. rerio* and reflected local river concentrations of the effluent (Castro et al., 2018). In addition, concentrations of 2% and 8% were also tested only in the calcium influx experiment in order to obtain a dose-response curve for the effluent. Effluent is a yellowish liquid. The chemical analysis was previously described by Castro et al. (2018): high amount of total phenols (3.8 mg/L) and acid resins (9.3 mg/L) and steroids below the detection limit.

### Animals

Experiments were conducted on male *Wistar* rats (*Rattus norvegicus*) at 10-day-old. Rats were obtained from the Central Animal House of the Federal University of Santa Catarina. They were bred in the animal house and maintained in an air-conditioned room (about 21 ± 2 °C) with controlled lighting (12/12 h light/dark cycle). The animals were fed pelleted food (Nuvital, Nuvilab CR1, Curitiba, Paraná, Brazil) and water was available *ad libitum*. All of the animals were carefully monitored and maintained in accordance with the local Ethical Committee for Animal Use (protocol CEUA-UFSC PP00862).

### Testes treatment

Rats were anesthetized with isoflurane by inhalation, then euthanized by decapitation and the testes were removed and decapsulated. Testes were incubated in Krebs Ringer-bicarbonate buffer (KRb) (122 mM NaCl, 3 mM KCl, 1.2 mM MgSO_4_, 1.3 mM CaCl_2_, 0.4 mM KH_2_PO_4_, 25 mM NaHCO_3_ and 5 mM glucose) or in KRb containing 4% effluent at pH 7.4 for 1 h at 34 °C.

### Primary culture of Sertoli cells

Sertoli cells were obtained from 10-day-old rat testes by sequential enzymatic digestion as previously described by Dorrington et al. (1975). Sertoli cells were seeded at a concentration of 650.000 cells/cm^2^ in 6 or 24-well culture plates. Four days after being plated, cells were used for the treatments with effluent and biochemical analyses.

### Measurement of total lactate content, LDH activity and glucose uptake

At the end of treatment, the incubation medium of testes was collected for determination of extracellular lactate content and extracellular LDH activity. Testes were homogenized, centrifuged and the supernatant was collected for intracellular lactate content and LDH activity by spectrophotometry based on the methods of Bergmeyer (1983) and Burtis et al. (2007), respectively. For glucose uptake, testes were incubated for 1 h in KRb buffer (control) or KRb supplemented with 4% effluent (treated), both containing 0.1 μCi/mL ^14^C-DG glucose. The radioactivity of testis homogenate was measured in a Tri-Carb 3180^®^ TR/SL spectrometer (Perkin-Elmer, Whaltam, MA, USA) (Kappel et al., 2013).

### Measurement of ROS, RNS and reduced glutathione (GSH) content and lipoperoxidation

Determination of ROS and RNS were based, respectively, on the oxidation of 2’,7’-dichloro-dihydro-fluorescein diacetate (H_2_-DCFDA) as described by Paraidathathu et al. (1992) and the oxidation of dihydrorhodamine 123 (DHR) as described by Ischiropoulos et al. (1999). GSH content was determined according to Beutler et al. (1963). The endogenous lipid peroxidation was evaluated in the testes by detection of substances that react with thiobarbituric acid (TBARS), according to the method described by Bird and Draper (1984). All of these assays were analyzed by spectrophotometry methods.

### Gamma Glutamyl Transpeptidase (GGT), Glutatione-S-Transferase (GST) and Superoxide Dismutase (SOD) enzyme activity

At the end of treatment, the testes were homogenized, centrifuged and the supernatant was collected for the GGT activity assay by spectrophotometry based on the methods of Orlowski and Meister (1963). The GST and SOD activity was measured in homogenate using the methodology described by Habig et al. (1974) and Misra and Fridovich (1972), respectively.

### Mitochondrial oxygen consumption of Sertoli cells

For the mitochondrial oxygen consumption assay, Sertoli cells were incubated in KRb buffer in the presence or absence of 4% effluent for 1 h. The respiration of the suspension of 350.000 cells/mL was measured at 34 °C by high-resolution respirometry using an Oroboros^®^ oxygraph (De Paula Martins et al., 2013). DatLab software (Oroboros Instruments, Innsbruck, Austria) was used for data acquisition (1 or 2 s intervals) and analysis. The experimental regime was started with ROUTINE state (respiration without additional substrates or effectors). After the ATP synthase activity was inhibited with oligomycin (1 μg/mL), in order to detect the level of LEAK stage (state of respiration independent of ADP phosphorylation, and mainly occurs due to proton leak from the mitochondrial inter-membrane space). The maximal capacity of the electron transport system (ETS stage) was obtained by uncoupling of oxidative phosphorylation by stepwise titration of FCCP (0.6 μM). Finally, addition of rotenone (0.5 μM) and antimycin A (2.5 μM) inhibited I and III complex activity. The resulting state provides a measure of residual oxygen consumption (ROX state) (Hall et al., 2013).

### Secretory activity of Sertoli cells

The procedures were performed according to Zanatta et al. (2013). Cells were incubated with fluorescent dye quinacrine (3 μM), which stains acidic vesicles, for 20 min at 34 °C. After cells were washed in Hank’s Buffered Salt Solution (HBSS) (16.7 mM, NaHCO_3_, 136.9 mM NaCl, 1.3 mM CaCl_2_, 5.4 mM KCl, 0.65 mM MgSO_4_, 0.27 mM Na_2_HPO_4_, 0.44 mM KH_2_PO_4_, 6.1 mM glucose), exposed to control solution (HBSS) or treatment solution (HBSS containing 4% effluent) and, immediately observed on an Olympus BX41 fluorescence microscope, using a green filter. Sequential images, every 2 min during 9 min, were obtained using the Qcolor 3C digital (Q-imaging) system (Espoo, Finland). Secretion was identified as the loss of fluorescent vesicles, indicating the fusion of secretory vesicles at plasma membrane. Percentage of the number of remaining vesicles *per* area *per* cell was quantified (using the program Image J^®^) and plotted on a graph as a function of time. The area under the curve was calculated in order to verify if there was difference between the group treated with effluent and the control group.

### Determination of calcium influx (^45^Ca^+2^) in the testis

To determine the time course of effluent effects on calcium influx, testes were incubated for 30, 300 and 900 seconds with 4% effluent or KRb. In order to evaluate the dose-response relationship testes were incubated for 900 seconds with KRb (control) or effluent at 2, 4 or 8%. To block calcium transport in the testes, a lanthanum buffer solution (127.5 mM NaCl, 4.6 mM KCl, 1.2 mM MgSO_4_, 10 mM HEPES, 10 mM LaCl_3_, 11 mM glucose, pH 7.4) was added to the incubation medium immediately after the incubation time. The testis homogenate radioactivity was measured in a Tri-Carb 3180® TR/SL spectrometer (Perkin-Elmer, Whaltam, MA, USA) (Rosso et al., 2012).

### Protein measurements

Protein was quantified using a standard concentration-curve with bovine serum albumin as reported by Lowry et al. (1951).

### Statistical analysis

For the testis experiments data were presented as the mean ± S.E.M. or percentage of control from 2 independent experiments with three-five animals in each group *per* experiment. For the cell experiments data were presented as the mean ± S.E.M. from two independent cultures. A pool of cells from 10 animals was used *per* culture. In experiments about cell secretion each analyzed cell was considered an “n”. The statistical analysis was performed with the Statistic 8 program by using Student's *t*-test for dependent or independent samples. Values ​​of p≤ 0.05 were considered significant.

## Results

### Effects of pulp mill effluent on energy metabolism in immature rat testis

**Figure 1:**
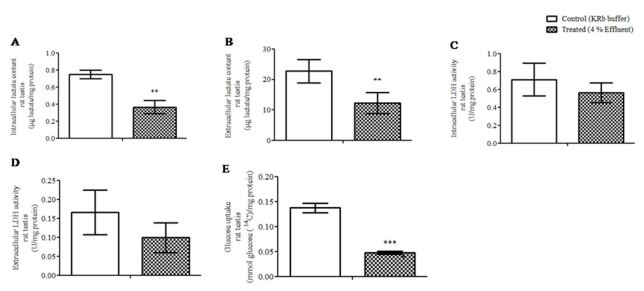
Changes in the energy metabolism of immature rat testes in response to a pulp and paper mill effluent. Testes were treated *in vitro* for 1 h with KRb as a control or 4% effluent. (A) Intracellular lactate content (n= 6); (B) extracellular lactate content (n= 8); (C) intracellular lactate dehydrogenase enzyme activity (n= 5); (D) extracellular lactate dehydrogenase enzyme activity (n= 6); and (E) glucose uptake (n= 5) Testis data were presented as the mean ± S.E.M. of 2 independent experiments with three-five animals in each group per experiment. Statistically significant differences from controls, as determined by Student’s *t*-test for paired samples (p≤ 0.05). **p≤ 0.01; ***p≤ 0.001. KRb: Krebs Ringer bicarbonate buffer.

Treatment *in vitro* of immature rat testes from 10-day-old with pulp and paper mill effluent for 1 h led to decreased intracellular lactate content (p≤ 0.01, Figure 1A) and secreted lactate (p≤ 0.01, Figure 1B). In order to determine if the decrease of lactate content was caused by a change in the activity of the enzyme that produces the lactate, the activity of the LDH enzyme was examined. The activity of intra- and extracellular LDH enzyme was reduced, but not statistically significant by 4% effluent in the testis from 10-day-old rats (Figure 1C and Figure 1D). We also evaluated whether the decrease in lactate content was associated with a change in glucose uptake. In this case, effluent treatment decreased significantly glucose uptake (p≤ 0.001) in the testes by about 65% (Figure 1E).

### Effects of pulp and paper mill effluent on oxidative metabolism in immature rat testis

**Figure 2:**
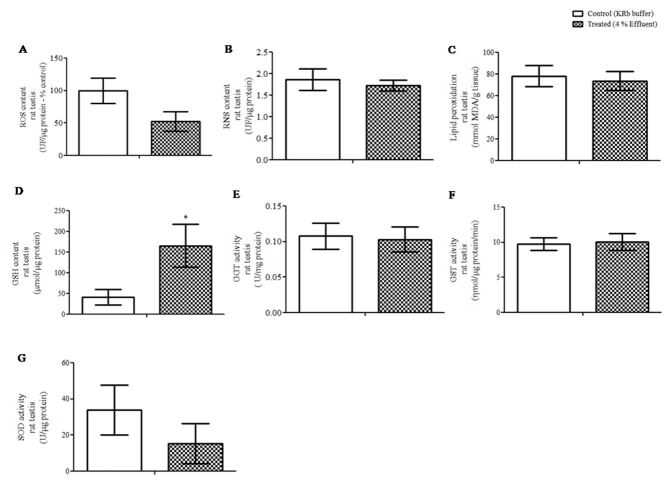
Oxidative metabolism in rat testes in response to pulp and paper mill effluent. Testes were incubated *in vitro* for 1 h with KRb (control) or 4% effluent (treated). (A) Reactive oxygen species (ROS) content (n= 5); (B) reactive nitrogen species (RNS) content (n= 10); (C) lipid peroxidation (n= 5); (D) reduced glutathione (GSH) content (n= 8); (E) gamma glutamyl transpeptidase (GGT) enzyme activity (n= 8); (F) glutathione S-transferase (GST) enzyme activity (n= 9); and (G) superoxide dismutase (SOD) enzyme activity (n= 5). Data were presented as the mean ± S.E.M. or percentage of control of 2 independent experiments with three-five animals in each group *per* experiment. *Statistically significant differences from controls, as determined by Student’s *t*-test for paired samples (p≤ 0.05). UF: units of fluorescence; KRb: Krebs Ringer-bicarbonate buffer; MDA: Malondialdeyde.

Figure 2 shows the action of effluent in the testis oxidative metabolism. In order to find some deleterious effect of the effluent on the testis, we initially evaluated whether the effluent could alter the content of reactive oxygen species, reactive nitrogen species and induce lipid peroxidation. It was verified that, at the selected time and concentration, these oxidative metabolism patterns were reduced, but not statistically significant (Figures 222C). In a second approach, we verified the status of the antioxidant defenses in the testis, under effluent action, through the content of GSH (Figure 2D) and GGT, GST and SOD enzyme activity (Figures 222G, respectively). The content of GSH was increased (p≤ 0.05) about 400%, compared to the control, while there were no alterations in the enzyme analyzed patterns.

### Effects of pulp and paper mill effluent on mitochondrial and secretory activity from Sertoli cells

**Figure 3:**
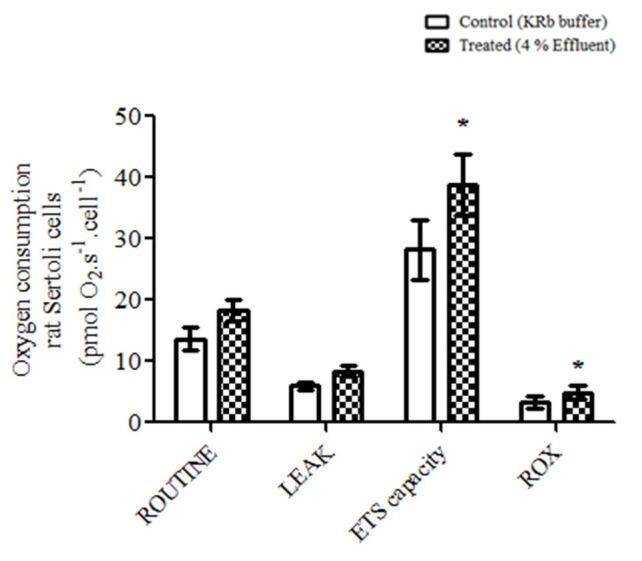
Effect of pulp and paper mill effluent on oxygen consumption by immature rat Sertoli cells. The mitochondrial oxygen consumption was evaluated at different states (ROUTINE, LEAK, ETS and ROX) after 4% effluent challenge by 1 h. Values are mean ± S.E.M. of five independent experiments (one determination of 350.000 cells per group per experiment). *Statistically significant differences from controls, as determined by Student's *t*-test for paired samples (p≤ 0.05).

The results in Figure 3 show how 4% effluent affected respiratory states in Sertoli cells. It is remarkable how the effluent tended to cause an increase in the oxygen consumption in all the stages of the mitochondrial respiration analyzed. However, the increase was statistically significant only in the maximal electron transport system (ETS) capacity (p≤ 0.01) and ROX (p≤ 0.01) states.

**Figure 4:**
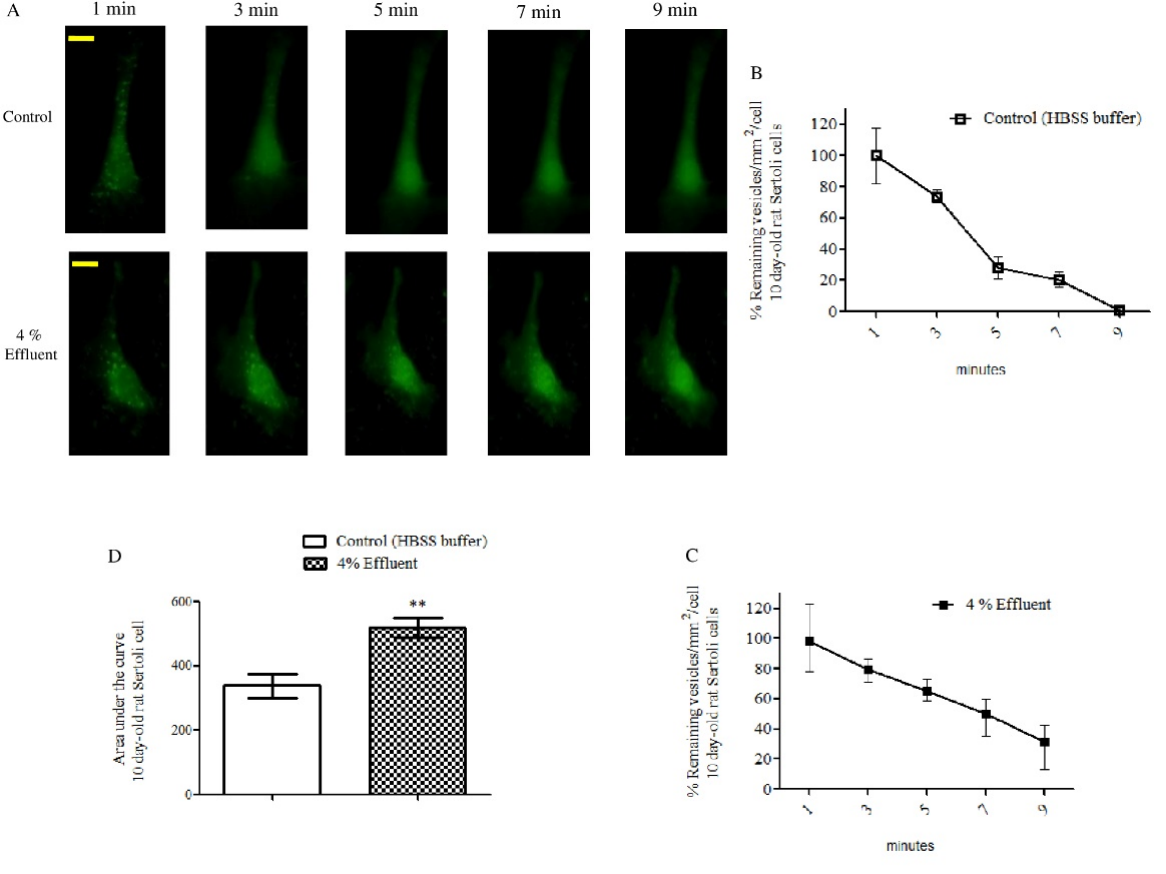
Effects of pulp and paper mill effluent on secretory activity by immature rat Sertoli cells. (A) Fluorescence images of Sertoli cells from 10-day-old rats stained with quinacrine and incubated *in vitro* with HBSS buffer (control) or 4% effluent. Percentage of number of vesicles per cell area as a function of time in the Sertoli cells incubated with HBSS buffer (B) or 4% effluent (C); (D) Area on the curve of both treatment groups. Cells in culture were incubated with 3 μM quinacrine, washed and photographed at 2 min intervals for 9 min of incubation with HBSS buffer (control) or 4% effluent (treated). Experiments were performed in 8 cells *per* group from two independent cultures (n= 8 individual cells) with similar results. For each Sertoli cell culture, a pool of 10 animals was used. Graph data were presented as the mean ± S.E.M. Statistical analysis was performed by using Student's *t*-test for impaired samples with **p≤ 0.01. HBSS: Hank’s Buffered Salt Solution. Yellow bar= 10 μm.

Figure 4 shows the result of Sertoli cells stained with quinacrine and then incubated with HBSS buffer or 4% effluent and immediately visualized in an inverted microscope. As seen in the representative images of the cells incubated with the treatments as a function of time (Figure 4A), the cells from rats of 10-day-old incubated with effluent took a longer time to lose the fluorescent vesicles. Figure 4B shows that after nine minutes of incubation the cells of the control group had lost 99% of their fluorescent vesicle content. At the same time, cells treated with 4% effluent lost 69% of the fluorescence vesicles. Difference between the two groups is statistically proved (p≤ 0.01) by the analysis of the area under the curve, shown in Figure 4C.

### Effects of pulp mill effluent on testis calcium homeostasis

**Figure 5:**
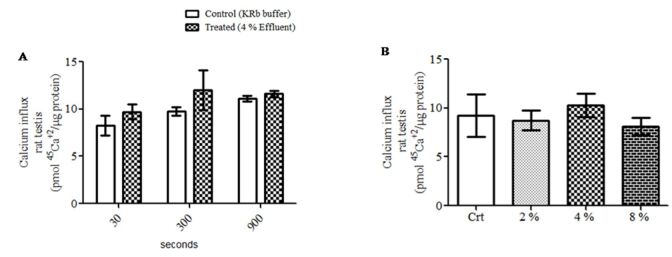
Calcium influx from immature rat testes on acute pulp and paper mill effluent treatment. (A) Time-course on 10 day-old rat testes (n= 5); (B) Dose-response curve in different concentrations (2%, 4%, 8%) on 10 day-old rat testes (n= 9). For the time-course, the testes were pre-incubated for 30 min with KRb buffer and then incubated for 30, 300 or 900 seconds with 0.1 µCi/mL of ^45^Ca^2+^ in the presence of KRb (control) or 4% effluent (treated). For the dose-response curve, the testes were pre-incubated for 30 min with KRb buffer and then incubated for 900 seconds with 0.1 µCi/mL of ^45^Ca^2+^ in the presence or absence of 2, 4 or 8% of effluent. Data were presented as the mean ± S.E.M. of 2 independent experiments with three-five animals in each group *per* experiment. Statistical analysis was performed by using Student's *t*-test for paired samples with p≤ 0.05. Crt: control; KRb: Krebs Ringer-bicarbonate buffer.

Figure 5 shows that calcium homeostasis in the testis of rats was neither affected by the acute effect of effluent at any of the incubation times (Figure 5A) nor at different concentrations of effluent tested (Figure 5B).

## Discussion

A decrease in lactate content in the testis of 10-day-old rats with pulp and paper mill effluent exposure is considered an important find since lactate is described as the main nutrient of spermatocytes and spermatids (Rato et al., 2012). Additionally, lactate is needed for RNA and protein synthesis stimulation in spermatids (Jutte et al., 1981) and exerts an antiapoptotic effect on germ cells (Erkkilä et al., 2002). These findings were consistent with our previous work in which male fish *D. rerio* exposed to 4% effluent for 14 days had decreased testis lactate content (Castro et al., 2018). Surprisingly, in the present study, the decrease in lactate content was not accompanied by the decrease in the activity of its synthesizing enzyme, LDH, which was unchanged. However, there was a significant decrease in glucose uptake in the testis, which may have caused the lactate content diminution. Since BTB regulates the passage of molecules (França et al., 2016) and that it is not yet established at 10-day-old (Morales et al., 2007) it could explain the vulnerability of the immature testes in the analyzed patterns. It is possible that effluent molecules interfere with glucose transporters (GLUTs) of Sertoli cells, which are mainly GLUT_1_, GLUT_2_ and GLUT_3_ (Galardo et al., 2008). A future analysis of these transporters is required.

Pro-oxidant pulp mill effluent activity in the testis has already been shown by Castro et al. (2018). No change in ROS, RNS and TBARS content parameters was different what expected in our initial hypothesis. It may be explained by the cellular characteristics of the rat testis at 10-day-old. Considering the cell types that make up the testis, Sertoli cells and spermatogonia are the least affected by oxidative stress as they have a complete and efficient antioxidant defense arsenal compared to germ cells, Leydig cells and sperm cells (Bauché et al., 1994; Celino et al., 2011). Testis of 10-day-old rats is basically composed of Sertoli cells and spermatogonia and this may be the reason that the testis had no alterations in the content of reactive species nor lipid peroxidation after incubation with effluent. In a second approach we investigated if components of the antioxidant system of the testis would be activated by acute exposure to the effluent. Then we found that the GSH content was increased in the testis incubated with 4% effluent. Thus, increased GSH appears to be an antioxidant defense mechanism activated by the testes cells in the presence of the effluent.

Studies that show the analysis of the mitochondrial metabolism of Sertoli cells through sensitive methods are scarce in the literature. With the exception of an earlier study by our group (Gonçalves et al., 2017), no other studies on the analysis of the mitochondrial oxidative metabolism of Sertoli cells through a high-resolution respirometry (OROBOROS) method, as used in the present study, were found. Our data show that Sertoli cells incubated 1 h with 4% effluent showed increased oxygen consumption at the maximum capacity of the ETS state, where they are challenged with sequential titration of FCCP decoupling. Increased oxygen consumption in the ETS state, compared to the ROUTINE state is expected to be observed (Hall et al., 2013), since the decoupling of the inner mitochondrial membrane causes an influx of protons into the matrix, undoing the electrochemical force responsible for the synthesis of ATP, which causes a higher compensatory oxygen consumption (Hutter et al., 2004). Our results show that treatment with effluent caused an increase in the ETS state compared to the control group. Therefore, we speculate that 4% effluent has a role like FCCP on the ETS components, that is, the effluent would be acting as a decoupling of the oxidative phosphorylation in the mitochondria of Sertoli cells. We also detected a significant increased effect of effluent in the ROX state. The residual activity in this state is usually represented by partial reduction of oxygen into reactive oxygen species (Gonçalves et al., 2017). In this case, effluent significantly increased the formation of ROS in 10-day-old rat Sertoli cells. Both alterations could be deleterious to the maintenance of the integrity and correct functioning of Sertoli cells, since ATP synthesis is compromised in a decoupling situation of oxidative phosphorylation and increased ROS production could lead to damage of cellular structures.

It is known that Sertoli cells contribute to the nutritional requirements of germ cells by the secretion of nutrients and metabolic intermediates (Rato et al. 2012). Sertoli cells are still responsible for the secretion of carrier proteins of metal ions and vitamins, growth factors and interleukins that are vital for the correct development of germ cells (Reis et al., 2015) and a fluid that lubricates the seminiferous tubules which is important for maintaining the spermatogenesis (Smith et al., 2012). According to Liu et al. (2016) the altered secretory function of Sertoli cells may be responsible for disturbed spermatogenesis. The results presented here show that the 4% effluent was able to alter the secretory activity of the Sertoli cells, through a delay in the secretion of acidic vesicles. Thus disturbances caused in the cellular secretion by the effluent could compromise the function of the testes of immature rats. However, further studies are needed in order to characterize the mechanism involved on the alteration of cell secretion in the presence of effluent.

Finally, final objective was to evaluate if the effluent was capable of altering the calcium ion homeostasis in the testis. Our group reported how xenobiotics at low concentrations, widely distributed in the environment, can alter calcium homeostasis in testis and Sertoli cells from immature rats (Cavalli et al., 2013; Gonçalves et al., 2018). Different from our initial hypothesis, pulp and paper mill effluent was not able to alter calcium homeostasis in the testes of immature rats at the concentrations and treatment times analyzed.

The Canoas River is inserted in the Atlantic Forest biome. The region is inhabited by endangered mammals that depend directly on river waters such as *Puma concolor* and *Leopardus pardalis* (Corrêa de Deus and Bagatini, 2016). In addition, there is intense human activity along its hydrographic basin of direct use of the waters as fishing, agriculture, industrial activity and leisure (Lopes et al., 2019) Thus, the probability of fauna and human populations coming into contact with pollutants present in the effluent is very high. Taking into account the conservation of the mammal species that live there and the possible harmful effect that the analyzed effluent can have on the health of the human populations that live near the Canoas River, these data are of great relevance.

## Conclusion

In a whole, our results showed that the pulp and paper mill effluent, in a short time of exposure and an environmentally relevant concentration, altered important metabolic functions involved on spermatogenesis in the testes of immature rats and in Sertoli cells. Further studies should determine whether the effluent also affects metabolic functions important to male fertility *in vivo* and in adult animals.
